# Manganese-enhanced T_1_ mapping to quantify myocardial viability: validation with ^18^F-fluorodeoxyglucose positron emission tomography

**DOI:** 10.1038/s41598-020-58716-x

**Published:** 2020-02-06

**Authors:** Nick Spath, Adriana Tavares, Gillian A. Gray, Andrew H. Baker, Ross J. Lennen, Carlos J. Alcaide-Corral, Marc R. Dweck, David E. Newby, Phillip C. Yang, Maurits A. Jansen, Scott I. Semple

**Affiliations:** 1 British Heart Foundation Centre for Cardiovascular Science, University of Edinburgh, Edinburgh, UK; 2 Edinburgh Preclinical Imaging, University of Edinburgh, Edinburgh, UK; 3 Department of Cardiology, Royal Infirmary of Edinburgh, Edinburgh, UK; 4 Department of Cardiology, Stanford University, Stanford, CA US

**Keywords:** Predictive markers, Cardiac regeneration

## Abstract

Gadolinium chelates are widely used in cardiovascular magnetic resonance imaging (MRI) as passive intravascular and extracellular space markers. Manganese, a biologically active paramagnetic calcium analogue, provides novel intracellular myocardial tissue characterisation. We previously showed manganese-enhanced MRI (MEMRI) more accurately quantifies myocardial infarction than gadolinium delayed-enhancement MRI (DEMRI). Here, we evaluated the potential of MEMRI to assess myocardial viability compared to gold-standard ^18^F-fluorodeoxyglucose (^18^F-FDG) positron emission tomography (PET) viability. Coronary artery ligation surgery was performed in male Sprague-Dawley rats (n = 13) followed by dual MEMRI and ^18^F-FDG PET imaging at 10–12 weeks. MEMRI was achieved with unchelated (EVP1001-1) or chelated (mangafodipir) manganese. T_1_ mapping MRI was followed by ^18^F-FDG micro-PET, with tissue taken for histological correlation. MEMRI and PET demonstrated good agreement with histology but native T_1_ underestimated infarct size. Quantification of viability by MEMRI, PET and MTC were similar, irrespective of manganese agent. MEMRI showed superior agreement with PET than native T_1_. MEMRI showed excellent agreement with PET and MTC viability. Myocardial MEMRI T_1_ correlated with ^18^F-FDG standard uptake values and influx constant but not native T_1_. Our findings indicate that MEMRI identifies and quantifies myocardial viability and has major potential for clinical application in myocardial disease and regenerative therapies.

## Introduction

Magnetic resonance imaging with gadolinium delayed-enhancement (DEMRI) has an increasingly important role in assessing myocardial viability by quantifying transmurality of scar^1,2^. With DEMRI, gadolinium partitions into the extracellular space including tissue with membrane disruption due to infarction and fibrosis. Although readily available clinically, DEMRI only describes infarcted or scarred tissue, from which viability is inferred. It cannot not directly assess viability, but does assess scar burden as a surrogate from which to predict the likelihood of functional recovery. Furthermore, long-term safety questions have recently been raised around some gadolinium formulations^3–5^. These limitations highlight the increasing need for novel cardiac MRI contrast media.

Manganese is a paramagnetic calcium analogue which is biologically active. It combines T_1_ shortening properties with intracellular uptake specifically defining myocardium with functional calcium-handling^6^. An essential trace element, manganese is fundamental to normal neurological, endocrine and bone development and function^7^. Applied to cardiac MRI, the combination of paramagnetism with uptake by functional calcium-channels offers highly attractive potential to selectively define regions of myocardium with functioning calcium-cycling. As well as defining viable tissue, there is scope to detect altered calcium-handling states observed in cardiomyopathies^8^. This technique has potential to add important functional assessment to current imaging with gadolinium delayed-enhancement which principally defines structure of pathology.

Unlike gadolinium, which has no known role in mammalian biology and is highly toxic in its unbound form^9^, manganese is naturally occurring with metabolic pathways for excretion providing a more attractive safety profile^10^. Manganese-based contrast media can be administered in chelated (e.g. mangafodipir, manganese dipyridoxyl diphosphate, Mn-DPDP), or non-chelated (e.g. EVP1001-1, manganese gluconate with calcium gluconate) formulations. Both formulations demonstrate robust safety profiles in clinical studies whilst retaining favourable imaging properties. Our previous preclinical work demonstrated both these agents can be used to achieve MEMRI T_1_ mapping, which quantifies infarct size more accurately than DEMRI and DEMRI T_1_ mapping, and has potential to track and quantify altered calcium-handling in remodelling myocardium^11^.

Glucose is the substrate for myocardial energy. The radio-labelled glucose analogue ^18^F-fluorodeoxyglucose (^18^F-FDG) is used routinely in nuclear imaging to track metabolism-dependent processes. Applied to cardiac imaging, ^18^F-FDG positron emission tomography (PET) is the gold standard for assessing myocardial viability^12,13^, offering the greatest sensitivity for viable myocardium and comparable specificity to other modalities^1^. Although^18^F-FDG PET necessitates exposure to radiation and its use is often limited by availability and expertise, imaging in this way directly assesses viability through metabolic functionality of tissues; a mechanistically similar method of myocardial viability assessment to MEMRI. Comparison of MEMRI with the gold-standard of ^18^F-FDG PET for myocardial viability assessment may therefore be of greater value than direct comparison with DEMRI techniques.

We aimed to compare and validate the quantification of viable myocardium by MEMRI T_1_ mapping using two distinct clinical grade agents and ^18^F-FDG PET in a rodent model of myocardial infarction. This novel comparison of MEMRI with ^18^F-FDG PET builds on previous preclinical work and is an essential mechanistic precursor to enable clinical translation of this contrast agent with notable potential for true viability assessment in cardiac MRI.

## Methods

All experiments were approved by the University of Edinburgh Animal Welfare and Ethical Review Body (713-LF2-16), carried out according to UK Home Office Animals (Scientific Procedures) Act 1986 (Project Licence 70/8933) and the European Parliament Directive on the protection of animals used for scientific purposes (2010/63/EU). Male Sprague Dawley rats (180–300 g, n = 16, Charles River Ltd, Haddington, UK) with free access to food and water and no dietary restrictions, were housed in the Bioresearch facilities, University of Edinburgh and allowed to acclimatise for 7 days prior to use in the study.

### Myocardial infarction model

Inhaled agents were exclusively used for anaesthesia, to allow rapid recovery in an effort to reduce peri-operative mortality, as advised by institutional veterinarians. Rats were anaesthetised with isoflurane (5% in 1.5 L/min oxygen) and maintenance anaesthesia (2–3% in 1 L/min oxygen). For analgesia, buprenorphine 0.05 mg/kg (Alstoe Ltd, York, UK) was administered before surgery and as required for 24 hours post-operatively. Anaesthetic depth was monitored throughout and deemed adequate by the absence of neurological response to painful physical stimulus. Using a bespoke intubation rig, the trachea was intubated under direct vision whilst maintaining inhaled nasal anaesthesia, whereby ventilation was subsequently established and maintained with a rodent ventilator (Harvard Apparatus Model 683, MA, USA, tidal volume 2.5 cm^3^, respiratory rate 60/min). Myocardial infarction was induced as previously described^11^. In brief, lateral thoracotomy was performed at the third or fourth intercostal space, the left lung collapsed and the pericardium ruptured. The heart was gently exteriorised and a non-absorbable 5-0 ligature placed around the left anterior descending coronary artery just above the bifurcation of the first diagonal, before being replaced in the thorax. Excess air and fluid were aspirated from the thorax as the wound was closed in three layers. Animals were weaned from inhaled anaesthesia over 5–10 minutes and extubated once spontaneous ventilation was re-established, housed at 30 °C for 24 hours and given sterile sodium chloride 0.9% 0.01 mL/g fluid therapy subcutaneously. After 24 hours, normal housing conditions were resumed.

### Magnetic resonance image acquisition and reconstruction

MRI with T_1_ mapping was performed using the methods previously described^11^. In brief, a 7 T horizontal bore NMR spectrometer (Agilent Technologies, Yarnton, UK), equipped with a high-performance gradient insert (120-mm inner diameter), maximum gradient strength 400 mT/m. Rats were anaesthetised using 1.5–2% isoflurane (Zoetis Ltd., London UK) in oxygen/air (50/50, 1 L/min), the tail vein cannulated and placed in a cradle (Rapid Biomedical GmbH, Rimpar, Germany). A 72-mm quadrature volume coil was used for transmission with signal reception by a four-channel phased array coil (Rapid Biomedical GmbH, Rimpar, Germany). Heart rate, respiration rate, and temperature were monitored (Model 1030 monitoring and gating system, Small Animal Instruments Inc., Stony Brook, NY, USA), maintained at 37 °C.

**Figure 1:**
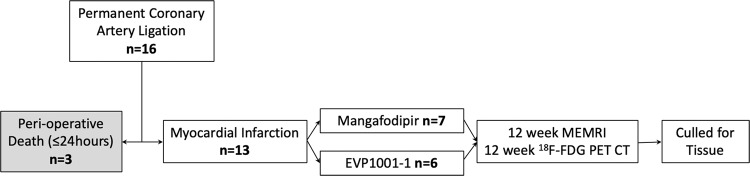
Experimental protocol. Flow chart of experimental protocol.

All imaging was performed 12 weeks after surgery (Fig. 1). This time point was chosen to assess established myocardial infarction with left ventricular remodelling, in line with our previous work in this field^11^. Cine imaging was acquired in long and short-axis stack orientation (9 × 2 mm slices) from the aortic valve annulus to the apex, perpendicular to the interventricular septum, using cardiac-gated gradient echo imaging (TR = 6.35 ms; TE = 1.4 ms; flip angle = 15°; FOV = 50 × 50 mm^2^; matrix = 128 × 128; slice thickness = 1.5 mm). The final slice plan and orientation was agreed and documented by two adjudicators (NS/MAJ) at the time of image acquisition to optimise repeatability between scans. Similarly, a single short-axis slice at the maximal infarct region was then agreed and the precise slice location recorded.

Native T_1_ mapping was performed for calculation of regional myocardial T_1_ relaxation times using a gradient-echo, cardiac-gated Modified Look-Locker Inversion recovery sequence (MoLLI). Images (n = 14–20) were acquired at inversion times determined by R-R interval, with the TR_inversion_ > 3xT_1_ of myocardium (TR_inversion_ > 4.50 s). Image readout was with a cardiac fast gradient echo (TR = 3.50 ms; TE = 1.77 ms; flip angle = 10°, matrix 128 × 128; Echo train length = 8; FOV = 50 × 50 mm^2^; in-plane resolution = 0.39 × 0.39 mm^2^; trigger delay = 1xR-R; slice thickness = 2 mm; 8 signal averages to compensate for respiratory motion).

MEMRI T_1_ mapping was achieved using either EVP1001-1 or mangafodipir (22 μmol/kg, n = 6 and 44 μmol/kg, n = 7 respectively), administered via slow intravenous injection into the tail vein over 1–2 min. Optimal dose and timings for post-contrast imaging differ between these agents, as we have previously demonstrated^11^. Optimal myocardial T_1_ shortening is achieved by 20 min for EVP1001-1 and from 40 min after administration of mangafodipir. T_1_ mapping was performed at the identical maximal infarct slice to native T_1_ mapping, as above.

### ^18^*F*-FDG PET/CT image acquisition and reconstruction

Maintained under inhaled anaesthesia, animals were transferred to a micro-PET/CT scanner (Nanoscan, Mediso Medical Imaging Systems, Hungary) whereby tail-prick blood glucose was measured prior to administration of intravenous ^18^F-FDG (20–30 MBq). PET data were acquired continually for 60 min immediately following intravenous radiotracer administration. A CT scan (semi-circular full trajectory, maximum field of view, 480 projections, 50 kVp, 300 ms and 1:4 binning) was acquired for attenuation correction. The emission dynamic 60-min scan was obtained using 3-dimensional 1:5 mode and re-binned as follows: 6 × 1 min; 7 × 2 min; 8 × 5 min. PET images were reconstructed using Mediso’s iterative Tera-Tomo 3D reconstruction algorithm and the following settings: 4 iterations, 6 subsets, full detector model, low regularization, spike filter on, voxel size 0.4 mm and 400–600 keV energy window. PET data were corrected for random co-incidents, scatter and attenuation.

### Image analysis

MRI image analysis was performed with identical methodology to previously described, including normalisation of T_1_ maps to the skeletal muscle for direct comparison of T_1_ values^11^. This was necessary due to high variation in T_1_ values measured before and after contrast due mainly to beat-to-beat variation in heart rate during prolonged anaesthesia. Baseline skeletal muscle T_1_ values for pre- (T_1bsm_) and post-contrast (T_1sm_) T_1_ maps were calculated from regions of interest (ROI) drawn in the chest wall in the intercostal muscles. The ratio of skeletal muscle T_1_ values (T_1bsm_/T_1sm_) was used to normalise the T_1_ maps at each time point using the equation nT_1*x,y*_ = T_1*x,y*_ x (T_1bsm_/T_1sm_) where T_1*x,y*_ is the absolute T_1_ of voxels within the ROI and nT_1*x,y*_ is the normalised T_1_ of voxels within the ROI.

The 14–20 images at unique inversion times were exported offline and incorporated with individual heart rate and trigger delay data using Matlab (MathWorks Inc., USA). T_1_ maps were then generated and analysed with commercially available software (CVI^4.2®^, Circle Cardiovascular Imaging, Calgary, Canada) using three-parameter non-linear curve fitting as previously described and standardized colour scales. Endocardial and epicardial contours drawn on MoLLI images were copied to the T_1_ map, once generated. Infarcted myocardium was defined as ≥ 2 x standard deviations of remote myocardial T_1_. Consecutive short-axis MRI cine slices were reconstructed to form a single 3D dataset [ImageJ, v1.51^14^] for co-registration with PET data.

PET data were analysed in the commercially available software (PMOD version 3.8, Fusion module, PMOD Technologies Limited, Zurich Switzerland), whereby PET and MR images were co-registered and fused. Endo-/epicardial contours were defined by MRI data with extent of PET uptake denoting myocardial viability. Presence of myocardium without PET uptake was defined as myocardial infarction, represented as a percentage short-axis slice corresponding to the T_1_ mapping slice. Rate of myocardial uptake ^18^F-FDG was then modelled using Patlak analysis^15^ to calculate influx constant (Ki) for remote myocardium (PMOD version 3.8, PMOD Technologies Limited, Zurich Switzerland).

### Histopathology

Animals were euthanised by overdose of isoflurane until circulatory arrest, followed by exsanguination. Hearts were fixed using immersion-fixation in 4% paraformaldehyde for 24 hours before transfer to 70% ethanol and processed to paraffin wax for sectioning. Serial 5-µm short-axis sections from apex to base were taken, registered to MRI short axis T_1_ map positions. Samples were stained with Masson’s trichrome (MTC) to delineate collagenous fibrosis, staining replacement scar tissue in the infarct blue and the non-infarcted myocardium purple, before mounting for computer-aided analysis. Slides were scanned at 20x magnification on a Zeiss Axioscan Z1 (Carl Zeiss AG, Oberkochen, Germany). Infarct size was calculated as a percentage of total left ventricular area at the corresponding maximal infarct slice as defined by MRI. Automated tissue detection was performed (Tissue Studio v2.4, Definiens AG, Munich, Germany) as previously described^11^ producing pixel counts and areas for the ROIs within the left ventricle, from which percentage infarct area at maximal infarct slice was calculated.

### Statistical analysis

Data are presented as mean ± standard deviation unless otherwise stated. Normality was assessed using the D’Agostino-Pearson test. For infarct quantification, native T_1_ mapping, MEMRI T_1_ mapping, PET non-viability and MTC assessments were compared using paired t-test and ANOVA (with Tukey’s multiple comparisons testing). Bland-Altman plots were used to assess agreement between native T_1_ mapping, MEMRI T_1_ mapping, PET non-viability and MTC. Spearman’s rank correlation tested for relationship between measures of ^18^F-FDG PET uptake (standardized uptake values [SUV] or influx constant [Ki], dependent variables) and remote myocardial indexed T_1_ values (native or post-MEMRI, independent variables). Statistical analysis was performed using GraphPad PRISM (v.7.0, GraphPad Software Inc., La Jolla, CA, USA). Statistical significance was taken as two-sided P < 0.05.

### Ethics

Ethical approval was granted for all animal studies by the University of Edinburgh Animal Welfare and Ethical Review Body (713-LF2-16), in accordance with the Animals (Scientific Procedures) Act, UK, 1986.

## Results

### Surgical outcomes

No animals died unexpectedly after recovering from surgery. Twelve weeks following surgery, all animals had left ventricular impairment with reduced ejection fraction (38.2 ± 10.2%). Whilst some variation in infarct size was seen, there were no differences between animals undergoing MEMRI with mangafodipir or EVP1001-1 in ejection fraction (39.2 ± 9.5% and 37.0 ± 10.8%, *P* = 0.69), end-diastolic volume (1.0 ± 0.2mls and 0.9 ± 0.2mls, *P* = 0.95) or left ventricular mass (0.7 ± 0.1 g and 0.7 ± 0.1 g, *P* = 0.40).

### Myocardial viability

**Figure 2:**
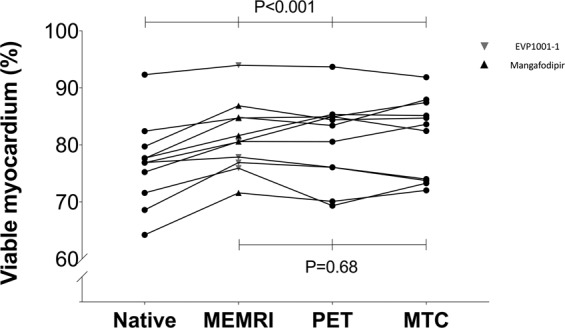
Viability quantification. Comparison of viability assessment by MEMRI, PET and MTC (n = 13). (MEMRI, manganese-enhanced magnetic resonance imaging; MTC, Masson’s trichrome; PET, positron emission tomography).

**Figure 3:**
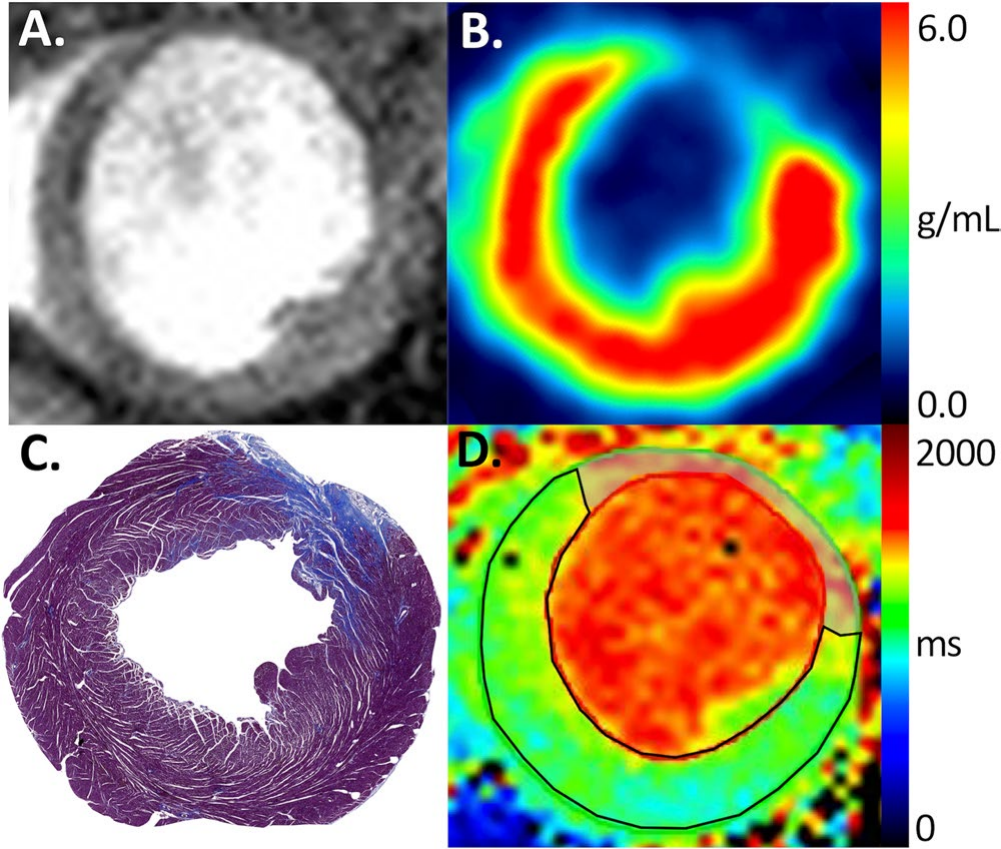
Representative ^18^F-FDG PET, T1 mapping and histopathology. End-diastolic cine image of left ventricular short axis slice (**A**) with co-registered ^18^F-FDG PET (**B**), MTC (**C**) and MEMRI T_1_ mapping (**D**). Anterior myocardial infarction is evident by the absence of ^18^F-FDG PET uptake in the anterior wall. Percentage of viable myocardium for ^18^F-FDG PET, MEMRI T_1_ mapping and MTC are calculated as 80.56%, 80.60% and 83.55% respectively. (^18^F-FDG, ^18^F fluorodeoxyglucose; MEMRI, manganese-enhanced magnetic resonance imaging; MTC, Masson’s trichrome; PET, positron emission tomography).

Quantification of viable myocardium was similar whether determined by PET, MEMRI T_1_ or MTC (Figs. 2 and 3), irrespective of manganese contrast agent (one-way ANOVA, Mangafodipir P = 0.12, EVP1001-1 P = 0.50). In contrast, viability quantification by native T_1_ mapping was lower than MEMRI T_1_ mapping (*P* < 0.01), PET (*P* < 0.01) or MTC (*P* = 0.03). Mangafodipir and EVP1001-1 independently demonstrated good agreement (Bland-Altman) with both PET (bias 0.22%, 95% CIs of LoA −3.69 to 4.12, P = 0.78 and bias 0.05%, 95% CIs of LoA −7.13 to 7.22, P > 0.9 respectively) and MTC quantification of viable myocardium (bias −1.41%, 95% CIs of LoA −5.79 to 2.94, P = 0.15 and bias 0.1.09%, 95% CIs of LoA −2.27 to 4.44, P = 0.18 respectively).

**Figure 4:**
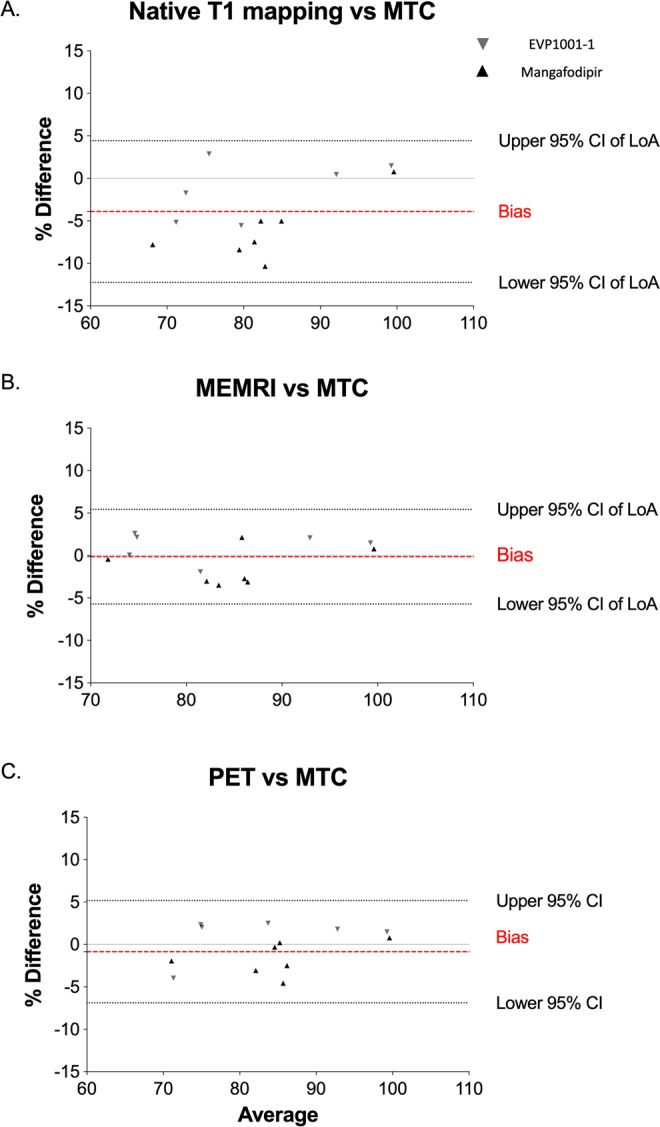
Comparisons with histopathology. Bland-Altman plots comparing viability by native T_1_ mapping (**A**, *P* = 0.03), MEMRI T_1_ mapping (**B**, *P* > 0.9) and ^18^F-FDG PET (**C**, *P* > 0.9) against histopathological comparator (MTC, all n = 13). (CI, confidence intervals; ^18^F-FDG, ^18^F fluorodeoxyglucose; LOA, limits of agreement; MEMRI, manganese-enhanced magnetic resonance imaging; MTC, Masson’s trichrome; PET, positron emission tomography).

**Figure 5:**
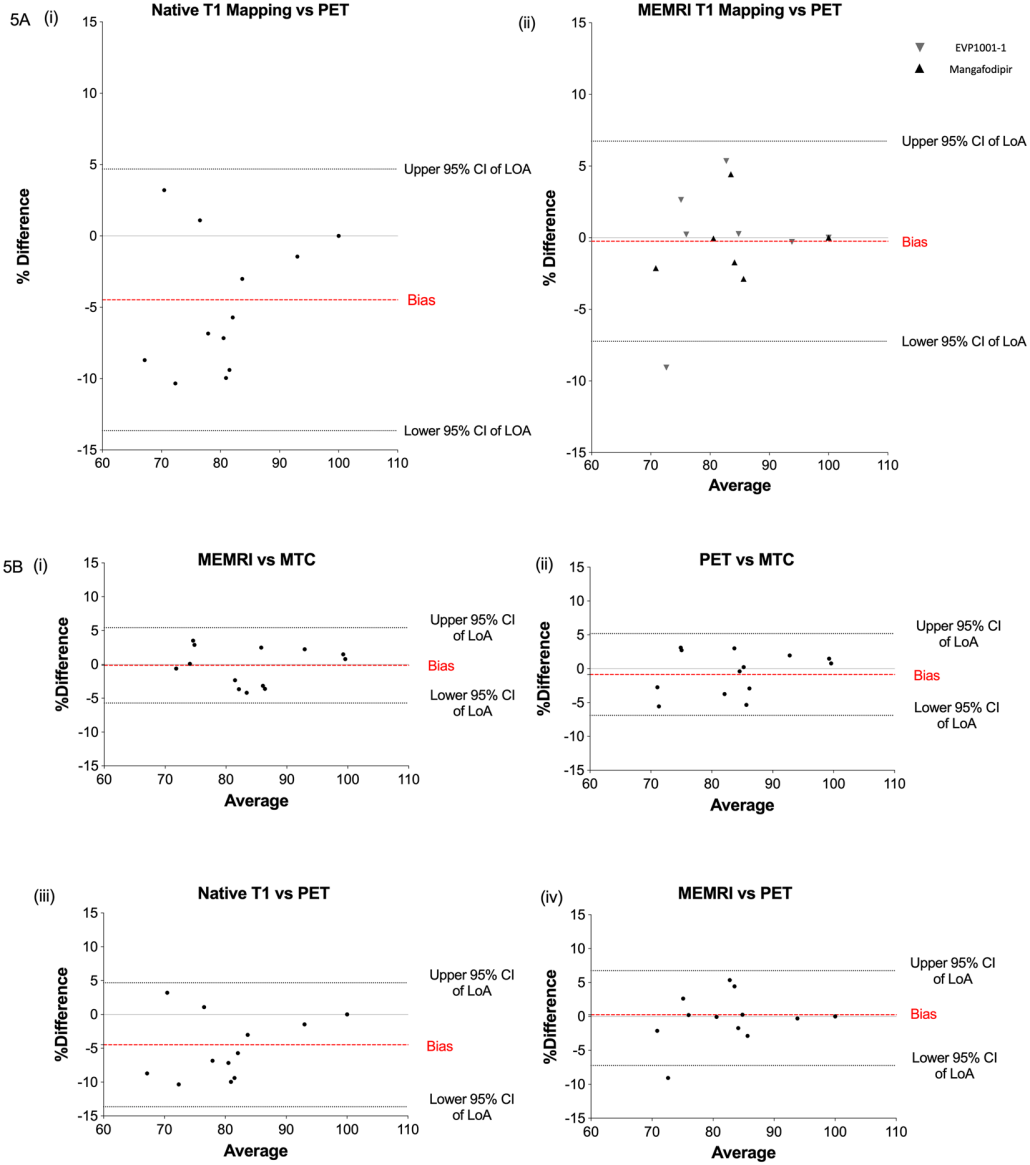
Comparisons with ^18^F-FDG PET. (**A**) Bland-Altman plots comparing viability by native T_1_ mapping and MEMRI T_1_ mapping against ^18^F-FDG PET (n = 13). (**B**) Bland-Altman plots comparing MEMRI T_1_ mapping (i) and ^18^F-FDG PET (ii) and histopathological comparator (MTC). Agreement between native T_1_ mapping (iii) and MEMRI T_1_ mapping (iv) with ^18^F-FDG PET (all n = 13). (CI, confidence intervals; ^18^F-FDG, ^18^F fluorodeoxyglucose; LOA, limits of agreement; MEMRI, manganese-enhanced magnetic resonance imaging; MTC, Masson’s trichrome; PET, positron emission tomography).

Bland-Altman analysis demonstrated excellent agreement of MEMRI T_1_ mapping and PET with MTC (bias −0.14%, 95% confidence intervals [CIs] of limits of agreement [LoA] −5.72 to 5.43, [P = 0.70] and bias −0.86%, 95% CIs of LoA −6.89 to 5.17 [P = 0.58] respectively), and this was superior to that of native T_1_ mapping, which tended to underestimate the amount of viable myocardium (bias −3.91%, 95% CIs of LoA −12.24-4.43, P = 0.006; Fig. 4). When compared to PET, MEMRI T_1_ mapping demonstrated superior agreement over native T_1_ mapping, which again underestimated viable myocardium (bias −0.25%, 95% CIs of LoA −7.23 to 6.74, *P* = 0.86, and bias −4.48%, 95% CIs of LoA -13.65 to 4.68, *P* 0.004, respectively; Fig. 5A). Strong agreement was observed between MEMRI T_1_ mapping (bias −0.14%, 95% CIs of LoA −5.72 to 5.43, *P* 0.70), as well as PET (bias −0.86%, 95% CIs of LoA −6.89 to 5.17, *P* = 0.58) and histological viability with MTC (Fig. 5Bi-ii). Similarly, with MEMRI T_1_ mapping and PET viability (bias 0.25%, 95% CIs of LoA −7.23 to 6.74, *P* = 0.86) although native T1 mapping tended to underestimate viable myocardium (bias −4.48%, 95% CIs of LoA −13.65 to 4.68, *P* = 0.004, Fig. 5Biii-iv).

### Manganese and ^18^F-FDG uptake

**Figure 6:**
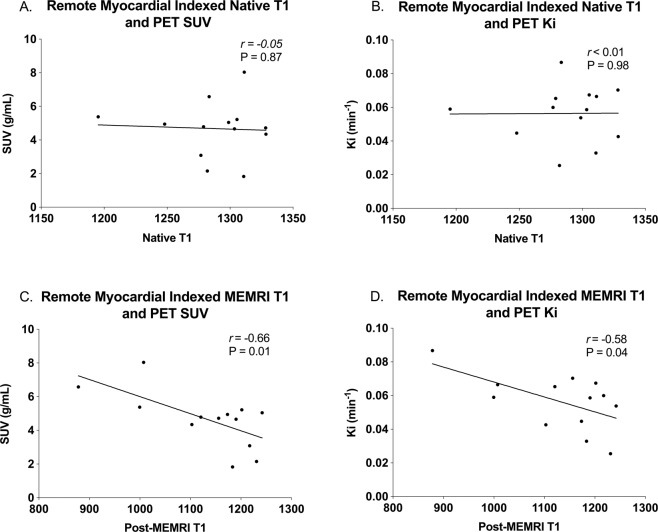
Myocardial ^18^F-FDG and manganese uptake. Native T_1_ values show no significant association with either measure of ^18^F-FDG PET uptake, SUV (**A**) or Ki (**B**), in contrast to MEMRI T1 values which correlate significantly with both (**C**,**D**, all n = 13). (^18^F-FDG, ^18^F fluorodeoxyglucose; Ki, influx constant; MEMRI, manganese-enhanced magnetic resonance imaging; PET, positron emission tomography; SUV, standardised uptake values).

On comparison of indexed T_1_ values with measures of ^18^F-FDG PET uptake, no correlation was evident between indexed native T_1_ mapping values and ^18^F-FDG PET SUV or Ki (*r* = −0.05, *P* = 0.87 and *r* < 0.01, *P* = 0.98). However, there was a negative correlation between MEMRI T_1_ mapping values and both ^18^F-FDG SUV and Ki (*r* = −0.66, *P* = 0.01 and *r* = −0.58, *P* = 0.04, Fig. 6).

## Discussion

In this study, we have validated cardiac MEMRI quantification using both ^18^F-FDG and MTC as imaging and pathologic gold-standard assessments of myocardial viability respectively. In addition, MEMRI T_1_ values demonstrated a relationship with ^18^F-FDG PET uptake suggesting both cellular calcium and glucose uptake are robust and concordant markers of myocardial viability. As a biologically active intracellular contrast agent, our study clearly demonstrates that MEMRI holds major potential to characterise myocardial structure and function that is readily translatable into clinical practice.

Several preclinical studies have used MEMRI to show successful engraftment of stem cells in the myocardium^16,17^. One clinical pilot study evaluated MEMRI in 10 patients following myocardial infarction, observing that regions remote to the infarct with preserved wall thickness were associated with increased manganese uptake^18^. However, this study did not use any other assessments of myocardial viability with which to compare the change in T_1_ signal. In the present study, we quantified viable myocardium with MEMRI T_1_ mapping which consistently demonstrated strong agreement with viability assessed by ^18^F-FDG PET, both of which were consistent with histopathological definition of viability. ^18^F-FDG PET defines viable myocardium by identifying myocardium with intact perfusion and preserved metabolism. We propose that MEMRI offers an MRI equivalent to this: an intracellular marker of viability that represents functional calcium-handling of contracting myocytes.

The question of what role non-invasive assessment of myocardial viability has in guiding revascularization therapies remains controversial and clinical trial data have yet to define its function in patient care. A substudy of the STICH trial^19^ looked for an association between myocardial viability quantification and clinical outcomes in patients undergoing revascularization or optimal medical therapy. Whilst observing no survival benefit in those with positive viability tests after adjustment for baseline variables, there were notable methodological shortcomings. Less than half of patients enrolled to this non-randomised substudy underwent viability testing and neither ^18^F-FDG PET or cardiac MRI were available at the time of enrolment. Furthermore, the viability assessments were dichotomised to presence or absence of viability only, without additional consideration of other important parameters of cardiac function. As such, the results of the study remain controversial. The AIMI-HF^20^ aims to provide definitive answers to the question of viability in ischaemic cardiomyopathy, and there remains a good rationale that non-invasive myocardial viability assessment may benefit certain well-defined patient groups in accurate prognostication and targeted therapy. Indeed, in a sub-group analysis of the PARR-2 trial^21^ where patients undergoing ^18^F-FDG PET-directed therapy derived significant benefit.

In clinical practice, characterizing myocardial scar by DEMRI is perhaps the most available non-invasive assessment of myocardial viability, with a transmural extent of infarct threshold of ≤50% denoting a >40% chance of improved contractility following revascularization, irrespective of degree of wall motion defect^2^. This technique has been well-validated and is considered a reference standard^22^. However, despite its high spatial resolution, assessing the extent of transmural viability is highly dependent on ability to comply with breath-holding, which can be challenging in complex co-morbid cardiac patients. Presence of myocardial oedema in conjunction with the timing of imaging is also crucial to avoid overestimation of infarct size which is recognised with DEMRI^23^. Moreover, predicting the probability of contractile recovery following revascularization depends on a close relationship of myocardial scar volume to viability, which may be an inferior surrogate to a direct measure of viable myocardial volume such as with ^18^F-FDG PET, to which MEMRI is mechanistically a paramagnetic equivalent.

Whilst nephrogenic systemic fibrosis has been virtually eliminated in modern day MRI, further safety concerns about gadolinium-based contrast media have arisen in recent years, predominantly over the discovery of gadolinium deposition in the basal ganglia with repeated exposure to linear chelates over time^3–5^. Although no definite pathological clinical sequelae have been identified to date, this has highlighted the current dependence on gadolinium-based contrast media and the increasingly urgent need for alternatives for paramagnetic or superparamagnetic magnetic resonance contrast imaging.

Of interest, the effect of manganese on T_1_ relaxivity correlated with both measures of ^18^F-FDG uptake. This indicates a key interrelationship between manganese and glucose uptake in viable cardiomyocytes, in remote as well as remodelling myocardium. Whilst it is not surprising that native T_1_ values showed no relationship with ^18^F-FDG PET, MEMRI T_1_ values demonstrated a significant correlation with both measures of radiotracer uptake. For remote viable myocardium with greater ^18^F-FDG uptake to exhibit an association with greater manganese uptake, as evidenced by lower MEMRI T_1_ values in these matched regions, supports the hypothesis that tracking functional calcium-handling is a relevant marker of myocardial viability. Successful clinical translation of this novel myocardial imaging modality offers exciting potential for MEMRI to non-invasively quantify viability without the use of ionising radiation. Clinical trial of MEMRI of the myocardium is ongoing at the time of writing, demonstrating feasibility of this modality in application to ischaemic and non-ischaemic cardiomyopathy (NCT03607669). If successful, detection of altered calcium-handling with MEMRI has potential utility in ischaemic as well as non-ischaemic myocardial insults such as dilated cardiomyopathy, tako-tsubo cardiomyopathy and hypertrophic cardiomyopathy.

It is important to acknowledge some limitations to our study. First, as the experimental protocol was designed to compare two intracellular viability agents, direct comparison of these data with gadolinium DEMRI techniques was not possible. However, the present study builds directly on our previous work which directly makes this comparison using an identical model and repetition of DEMRI was not considered ethically or scientifically justified^11^. Second, the use of a permanent coronary artery ligation model imaged at 12 weeks, may not reflect clinical disease as accurately as a reperfusion model, and longitudinal analysis limits the scope of our study. However, as we have previously published^11^, this model was chosen to result in myocardial infarction with a degree of left ventricular remodelling, providing a stable injury pattern from which robust comparisons can be drawn. Despite some variation in infarct size, all animals were included in the final analysis irrespective of viable myocardial percentage as each animal acted as its own control, demonstrating the ability of MEMRI to quantify myocardial viability in both extensive and subtle injury patterns. Finally, several additional challenges inherent to preclinical *in vivo* imaging (prolonged free-breathing T_1_ mapping sequences, variable and high heart and respiratory rates) with the lack of clinically available motion correction algorithms and small overall myocardial volume may have resulted in greater variability in the data, requiring normalisation. These challenges are not anticipated in clinical translation.

## Conclusion

For the first time, the present study validates the direct quantification of myocardial viability by MEMRI against ^18^F-FDG PET imaging. Whilst ^18^F-FDG PET is the clinical gold-standard, it is limited by radiation exposure and the lack of dynamic functional and structural assessment that is made possible by MRI. In contrast, MEMRI adds quantification of intracellular calcium-handling to the already comprehensive single-point assessment of cardiac MRI. This technique has major potential for clinical application in the assessment of myocardial viability in ischaemic cardiomyopathy as well as non-ischaemic cardiomyopathy and regenerative myocardial therapies. Clinical translation of myocardial MEMRI is an essential next step and studies should focus on further characterisation of uptake in healthy volunteers before applying MEMRI in patients with myocardial infarction and viability in ischaemic cardiomyopathy. Thereafter, application in non-ischaemic cardiomyopathy and assessments of regenerative therapies will be of major interest.

## Data Availability

The datasets generated and/or analysed during the current study may be available from the corresponding author on reasonable request.
